# Biosynthesis of quebrachitol, a transportable photosynthate, in *Litchi chinensis*

**DOI:** 10.1093/jxb/erx483

**Published:** 2017-12-21

**Authors:** Zi-Chen Wu, Jie-Qiong Zhang, Jie-Tang Zhao, Jian-Guo Li, Xu-Ming Huang, Hui-Cong Wang

**Affiliations:** 1 Guangdong Litchi Engineering Research Center, College of Horticulture, South China Agricultural University, Guangzhou, China; 2 Department of Life Sciences and Technology, Yangtze Normal University, Fuling, China

**Keywords:** Bornesitol, inositol methyltransferase, *Litchi chinensis*, osmotic stress, phloem transportation, quebrachitol

## Abstract

Although methylated cyclitols constitute a major proportion of the carbohydrates in many plant species, their physiological roles and biosynthetic pathway are largely unknown. Quebrachitol (2-*O*-methyl-chiro-inositol) is one of the major methylated cyclitols in some plant species. In litchi, quebrachitol represents approximately 50% of soluble sugars in mature leaves and 40% of the total sugars in phloem exudate. In the present study, we identified bornesitol as a transient methylated intermediate of quebrachitol and measured the concentrations of methyl-inositols in different tissues and in tissues subjected to different treatments. ^14^CO_2_ feeding and phloem exudate experiments demonstrated that quebrachitol is one of the transportable photosynthates. In contrast to other plant species, the biosynthesis of quebrachitol in litchi is not associated with osmotic stress. High quebrachitol concentrations in tissues of the woody plant litchi might represent a unique carbon metabolic strategy that maintains osmolality under reduced-sucrose conditions. The presence of bornesitol but not ononitol in the leaves indicates a different biosynthetic pathway with pinitol. The biosynthesis of quebrachitol involves the methylation of myo-inositol and the subsequent epimerization of bornesitol. An inositol methyltransferase gene (*LcIMT1*) responsible for bornesitol biosynthesis was isolated and characterized for the first time, and the biosynthesis pathways of methyl-inositols are discussed.

## Teaser

We report the involvement of quebrachitol in photosynthate transport and stress response, and characterize an inositol methyltransferase gene involved in the biosynthesis of bornesitol, the methylated intermediate of quebrachitol.

## Introduction

**Fig. 1.**
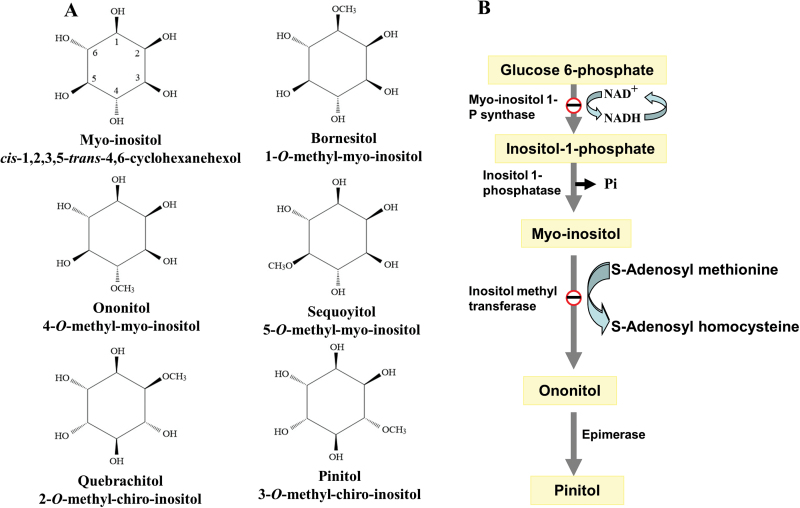
(A) The structural formulas of myo-inositol and five reported methyl-inositols. (B) Scheme of the pinitol biosynthesis pathway in plants. The open circle with a bar represents a rate-limiting reaction.

Sucrose is the major photosynthetic product and the predominant phloem-translocated compound in most plants (Zimmermann, 1960; Rennie and Turgeon, 2009). However, many plant species also synthesize sugar alcohols in their source leaves and translocate them to their sink organs (Loescher and Everard, 2000; Noiraud *et al.*, 2001*a*). Examples of such compounds include sorbitol, which is transported in members of the Rosaceae and Plantaginaceae; mannitol in members of the Apiaceae, Combretaceae, Oleaceae, and Plantaginaceae; and galactitol in members of the Celastraceae (Noiraud *et al.*, 2001*a*). In addition, the cyclitol compound myo-inositol is present in all plant species. Myo-inositol can be used as a substrate for the production and accumulation of methylated derivatives (Fig. 1A) such as bornesitol (1-*O*-methyl-myo-inositol), ononitol (4-*O*-methyl-myo-inositol), sequoyitol (5-*O*-methyl-myo-inositol), quebrachitol (2-*O*-methyl-chiro-inositol), and pinitol (3-*O*-methyl-chiro-inositol) (Loewus and Dickinson, 1982; Bieleshi and Briggs, 2005). These cyclitols have been found in taxonomically diverse species such as Chinese yew (*Taxus chinensis*), ice plant (*Mesembryanthemum crystallinum*), pigeon pea (*Cajanus cajan*), halophytic wild rice (*Porteresia coarctata*), *Aspidosperma quebracho*, *Hevea brasiliensis*, and *Ginkgo biloba* (Plouvier, 1960; Vernon *et al.*, 1993; Wanek and Richter, 1997; Sengupta *et al.*, 2008; Negishi *et al.*, 2015). Cyclitols are highly soluble and relatively metabolically inert (Loewus and Loewus, 1980), which allows them to accumulate to high levels without interfering with cellular structures or metabolism.

Pinitol, the best studied methyl-inositol, is a major carbohydrate in some plant species and, in some cases, can be more abundant than sucrose (Smith and Phillips, 1982; Chiera *et al.*, 2006). Guo and Oosterhuis (1995) showed that pinitol is a major transport carbohydrate in soybean (*Glycine max*), where it is mainly synthesized in mature leaves, although levels increase in all organs in response to high-temperature stress. In addition, a feeding experiment demonstrated that pinitol is directly unloaded from the seed coat before being transported to the embryo (Gomes *et al.*, 2005). Pinitol concentrations in soybean were also reported to increase 2- to 3-fold from the basal to the apex leaves of the plant; however, the difference in pinitol concentrations did not correlate with the activity of the key biosynthetic enzyme, inositol methyltransferase (Streeter *et al.*, 2001). This was interpreted as suggesting that pinitol levels reflected the translocation of cyclitol from the lower to the upper leaves. The current understanding of cyclitol transport in plants is still very limited, especially compared with knowledge of the transport of other polyols, such as sorbitol or mannitol (Noiraud *et al.*, 2001*b*; Reidel *et al.*, 2009). For example, the mechanisms of cyclitol transport within the plant have not been clearly demonstrated; however, the presence of cyclitols in phloem sap is indicative of their long-distance transport (Campbell and Binder, 1984).

Quebrachitol was first described as a natural product in *Aspidosperma quebracho* (Apocynaceae), and was later detected in various species of the Sapindaceae, as well as members of the Aceraceae and the Euphorbiaceae families (Díaz *et al.*, 2008). Within the Euphorbiaceae, the rubber tree species *Hevea brasiliensis* accumulates particularly high levels of quebrachitol, which is the predominant carbon compound in latex, representing approximately 1.2 % (w/v), in contrast to sucrose, which is present at 0.4 % (w/v) (Dusotoit-Coucaud *et al.*, 2010). A putative quebrachitol transporter, *HbPLT2*, was cloned and characterized in *H. brasiliensis* (Dusotoit-Coucaud *et al.*, 2010).

Litchi (*Litchi chinensis* Sonn.) is an important Sapindaceae fruit crop that is widely cultivated in warm regions of the world. High levels of quebrachitol have been detected in its leaves, bark, and fruit (Wang *et al.*, 2013). Quebrachitol is a less well studied methyl-inositol than pinitol and its physiological roles in plants have not been well defined, although cyclitols have been suggested to be major contributors to osmotic pressure (Richter and Popp, 1992; Streeter *et al.*, 2001; Sengupta *et al.*, 2008) and to act as cryoprotective agents (Orthen and Popp, 2000). The physiological roles of quebrachitol, whether it is a phloem transport photosynthate or a metabolic end-product, and how it is metabolized remain open questions.

The biosynthesis of pinitol is now well established (Fig. 1B). Myo-inositol is the precursor for pinitol synthesis and is synthesized by myo-inositol phosphate synthase (EC 5.5.1.4) and myo-inositol monophosphatase (EC 3.1.3.25) (Hegeman *et al.*, 2001; Styer *et al.*, 2004). Furthermore, a two-step pathway involving the methylation of myo-inositol by myo-inositol 4-*O*-methyltransferase (EC 2.1.1.129) and the subsequent epimerization of ononitol, the methylated intermediate, is the key mechanism for the production of pinitol (Loewus and Dickinson, 1982; Dittrich and Korak, 1984). Vernon and Bohnert (1992) identified a gene encoding a methyltransferase (*McIMT1*), which was shown to mediate a key step in stress-induced pinitol accumulation in *M. crystallinum*. Transgenic tobacco (*Nicotiana tabacum*) plants expressing *McIMT1* accumulated ononitol, which was not detectable in non-transformed control plants (Vernon *et al.*, 1993). Moreover, in transgenic soybean overexpressing *McIMT1*, ononitol levels were 10- to 80-fold higher than in non-transgenic tissues (Chiera *et al.*, 2006). These results indicate that ononitol is a stable intermediate in pinitol biosynthesis. In addition to inositol 4-*O*-methyltransferase, which catalyzes the formation of 4-*O*-methyl-myo-inositol (ononitol), multiple inositol methyltransferases have been identified. Inositol 3-*O*-methyltransferase (EC 2.1.1.39) and inositol 1-*O*-methyltransferase (EC 2.1.1.40) catalyze the formation of 3-*O*-methyl-myo-inositol and 1-*O*-methyl-myo-inositol (bornesitol), respectively (Schomburg *et al.*, 2006). Thus, the enzyme involved in the first step of quebrachitol biosynthesis might differ from that of pinitol biosynthesis.

The main aim of the current study was to elucidate the physiological roles and biosynthetic pathway of quebrachitol in litchi. To this end we evaluated the concentrations of quebrachitol in different tissues, as well as in phloem exudate, and in tissues subjected to osmotic stress. An *IMT* gene encoding an inositol methyltransferase was isolated and characterized, and the function of this gene was verified *in vivo* and *in vitro*. Here, we propose a draft outline of the biosynthetic pathway of quebrachitol and the involvement of quebrachitol in photosynthate transport and stress responses.

## Materials and methods

### Plant materials and treatments

The experiments were conducted at the experimental orchard of the South China Agricultural University, Guangzhou, China. The litchi (cv. Feizixiao) trees used for the experiment received standard horticultural practices, including disease and pest control. Mature leaves and stems from mature autumn shoots and roots were collected on October 15 2013, and mature fruits were collected on June 9 2014. Phloem and xylem tissues were dissected from the stem samples using a razor and the fruits were dissected to isolate the pericarp tissue, arils, seed coats, and cotyledons.

Autumn shoots were selected to investigate methyl-inositol concentrations during leaf development. Samples were collected from leaflets that had reached 2–3 cm in length (1 week after flushing) and every 7 days until full maturity, when leaf greenness (SPAD values) became relatively stable. SPAD values were measured before sampling, using a portable SPAD-502 chlorophyll meter (Minolta Camera Co. Ltd, Japan). Mature leaves from summer shoots were randomly chosen and leaf discs (≈1 cm^2^) were collected using a cork bore at dawn (before sunrise) and at dusk (before sunset) to monitor the diurnal concentrations of sucrose, methyl-inositols, and starch.

Sixteen potted litchi (cv. Feizixiao) trees were selected as material to investigate the effects of prolonged periods of darkness on leaf sucrose and methyl-inositol concentrations. Double-layer kraft paper was used to bag the shoots for 1 week; trees not subjected to bagging were used as controls. All plants were grown in a greenhouse at 28 °C using natural light with photon flux densities of ~800–1000 μmol m^–2^ s^–1^ at day length about 12 h. Leaf discs were sampled.

Fifteen 5-month-old plantlets were grown at 25 °C under conditions of 10 h light/14 h dark and ~50% relative humidity with photon flux density ~800 μmol m^–2^ s^–1^, before being separated into three groups. Five pots were watered with 400 mM NaCl every 3 days for 1 week; five pots were well watered with tap water (control); and the other five received a drought treatment with relative soil moisture content maintained at ~40%, monitored with a soil tensiometer. Leaf samples were collected 1 week after the stress or control treatment period.

Seeds of a large-seeded litchi cultivar (‘Shuilin’) were sown in acid-washed sand (pH 6.2) at a spacing of 5 × 10 cm. Seeds were sampled on the day of sowing, and seedlings were subsequently sampled at different developmental stages. The samples were washed with distilled water, freeze-dried, and ground in a pestle and mortar for analysis of quebrachitol and bornesitol contents.

Twenty mature shoots from different positions in the canopy, each with more than 10 leaves, were selected from a tree. They were randomly allocated to two treatment groups: girdling at a width of 5 mm at ~10 cm from the lowest leaf, and no girdling (control). Phloem samples above the girdle and the corresponding part from the control shoots were taken 4 weeks after girdling.

Eight replicates were used for leaf samples, while five replicates were used for the other samples. All samples were immediately frozen in liquid nitrogen and stored at –80 °C until use.

### Collection of phloem exudates

Mature leaves were excised by cutting the petioles 4–7 cm from the leaf blade while they were submerged in 5 mM 2-Na-ethylenediaminetetra-acetate (EDTA), pH 7.0. Fifteen minutes later, the petioles of three excised leaves were inserted into 1.5 ml Eppendorf tubes with 1.0 ml EDTA solution. The tubes with petioles were then placed in a closed chamber under low-light conditions and ~100% humidity to reduce transpiration, and the petioles were transferred to new tubes, containing fresh EDTA solution, every 2 h (Liu *et al.*, 2012).

### ^14^CO_2_ feeding assay

The ^14^C-labeling experiment was performed using mature leaves. Leaves were placed into a polyethylene chamber containing 480 μCi ^14^CO_2_ (generated from Ba^14^CO_3_) for 2 h. Shoots were then cut and soluble sugars in leaf and phloem were extracted and analyzed using HPLC as described by Wang *et al.* (2015). The eluates at 3.0–4.3 min (background), 7.2–8.2 min (sucrose), and 10.5–12.3 min (quebrachitol and/or bornesitol) were collected using an Agilent HPLC 1200 equipped with a fraction collector. The radioactivity in the eluates was measured using a liquid scintillation counter (Beckman Instruments Inc., Fullerton, CA, USA).

### Characterization of methyl-inositol and determination of carbohydrates

Non-structural carbohydrates were extracted and analyzed according to Wang *et al.* (2015). The presence of quebrachitol, initially suggested by the retention time under HPLC, was further confirmed by GC-MS as described by Wu *et al.* (2016). An unknown methyl-inositol with a different retention time from the methyl-inositol standards used was detected in leaf extracts under GC-MS equipped with a DB-35MS column (30 m × 0.25 mm × 0.25 μm). This fraction was separated using a Coregel 87C column (CHO-99–5860) and collected using an HPLC system (Agilent Technologies), freeze-dried, and redissolved in 1 ml of 99.96% D_2_O for NMR analysis in a Bruker AVANCE 600 MHz spectrometer, incorporating a 5 mm Broadband Observe probe, with tetramethylsilane as reference for the calibration spectrum (δ=0). All analyses were performed at 30 °C, with a frequency of 600 MHz for ^1^H NMR and 150 MHz for ^13^C NMR. Quebrachitol, pinitol, and sequoyitol were supplied by Sigma. Ononitol was purchased from Reagent Science Industry Ltd Co., New Zealand. Quantification of sucrose and quebrachitol was performed according to external standard solution calibrations. The unknown methyl-inositol was quantified using the standard curve of quebrachitol.

### Isolation and cloning of an inositol methyltransferase gene

Twenty-three nucleotide sequences that were annotated as putative methyltransferases and clustered with *IMT1* from ice plant (*McIMT1*) under phylogenetic analysis based on a multiple sequence alignment were obtained from a *L. chinensis* genome database (http://litchidb.genomics.cn/page/species/index.jsp). The expression patterns of these sequences in different organs were analyzed using quantitative real-time PCR (qRT-PCR) as previously described (Lai *et al.*, 2016). Gene-specific qRT-PCR primers (see Supplementary Table S1 at *JXB* online) were designed using Primer 5.0 (PREMIER Biosoft International, Canada).

Four sequences (Litchi_CLEAN 10022421, 10006652, 10040979, and 10040977) that showed high similarity to *McIMT1* and were specifically expressed in litchi leaves were selected for further study. The coding sequences of the putative *IMTs* were amplified using specific primers from a cDNA library, which reverse transcribed from leaf RNA.

### Heterologous expression of putative litchi *IMT* genes in *Escherichia coli*

The full-length coding sequences of *LcIMT1-4* (Supplementary Table S2) were cloned into the plasmid pET32a (Novagen). Overexpression of LcIMT1-4 was separately induced in *Escherichia coli* (DE3) by 0.3 mM isopropyl β-D-thiogalactoside when the cell density quantified by OD_600_ reached 0.6. The *E. coli* cells were grown in 1 l flasks with 500 ml Luria-Bertani medium. The recombinant His-tagged proteins were purified with a High Affinity Ni-NTA (Sangon Biotech Shanghai Co.) gravity-flow column. Ten column volumes of resin-bed elute buffer (20 mM Tris-HCl, 500 mM NaCl, 500 mM imidazole, pH 8.0) was applied and flow-through fluid collected. Each volume was quantitatively analyzed and the highest protein content was used in the next step. Proteins that eluted from the resin were separated by 12% SDS-PAGE analysis after Coomassie blue staining.

### Enzyme activity measurements

Methyltransferase activity of the purified recombinant proteins was detected as previously described (Vernon and Bohnert, 1992). The reaction mixture (200 µl) contained 50 mM Tris-HCl (pH 8.0), 10 mM MgCl_2_, 1 mM myo-inositol, and 100 μg purified recombinant protein. The reaction mixture was incubated at 30 °C for 15 min; *S*-adenosylmethionine was then added to a final concentration of 0.5 mM and the reaction mixture was incubated at 30 °C for 2 h. A reaction mixture without the addition of *S*-adenosylmethionine served as the control. The GC-MS conditions used to detect the formation of bornesitol were as described above.

### Virus-induced gene silencing of *LcIMT1* expression

Virus-induced gene silencing (VIGS) was carried out as previously described (Li *et al.*, 2016). The TRV1 vector and the TRV2 vector with a 300 bp *LcIMT1* fragment insert were transformed separately into *A. tumefaciens* strain GV3101 and then grown overnight in Luria-Bertani liquid medium containing 50 mg l^–1^ kanamycin and 1 mg l^–1^ rifampicin. *Agrobacterium* strains containing TRV1 and either TRV2-*LcIMT1* or empty TRV2 vector (control) were then mixed in a 1:1 ratio and injected evenly into litchi leaves still attached to the plant. Silencing of *Lc-PDS* was performed according to Li *et al.* (2016) in parallel to test the assay system. Samples were collected approximately 12 days after injection.

### Sequence analysis

Multiple sequence alignment was performed using ClustalX 1.83 (http://www.ebi.ac.uk) and MEGA5 (Tamura *et al.*, 2011).

## Results

### Presence of bornesitol in litchi

Previously, quebrachitol was detected as the major soluble carbohydrate in all tissues of litchi (Wang *et al.*, 2013). In the present study, an unknown peak with almost the same retention time as ononitol was observed in leaf extracts analyzed under a HPLC-refractive index detector (Supplementary Fig. S1). Although this compound (peak 5) displayed an identical mass spectrogram to that of other known methyl-inositols, the retention time (19.986 min) was longer than that of pinitol (17.823 min), quebrachitol (18.116 min), sequoyitol (18.346 min), and ononitol (19.202 min), suggesting that it was a different methyl-inositol (Supplementary Fig. S2). This compound was isolated and subjected to NMR analysis. ^1^H-NMR spectra identified nine hydrogen resonances and ^13^C-NMR spectra identified seven carbon resonances for the unknown putative methyl-inositol. The NMR spectra [^1^H-NMR (D_2_O, 600 MHz) *δ*:4.16 (1H, t, *J*=2.8 Hz, H-2), 3.49 (1H, m, H-6), 3.47 (1H, m, H-4), 3.36 (1H, dd, *J*=10.0, 2.9 Hz, H-3), 3.29 (3H, s, -OCH_3_), 3.15 (1H, t, *J*=9.4 Hz, H-5), 3.05 (1H, dd, *J*=10.0, 2.8 Hz, H-1); ^13^C-NMR (D_2_O, 150 MHz) *δ*:80.8 (C-1), 74.7 (C-5), 72.6 (C-4), 71.9 (C-6), 71.4 (C-3), 68.0 (C-2), 57.1 (-OCH_3_)] agreed with published spectra for bornesitol (1-*O*-methyl-myo-inositol) (Obendorf *et al.*, 2005). Thus, the compound was identified as bornesitol.

### Quebrachitol and bornesitol are present in different litchi tissues

**Fig. 2.**
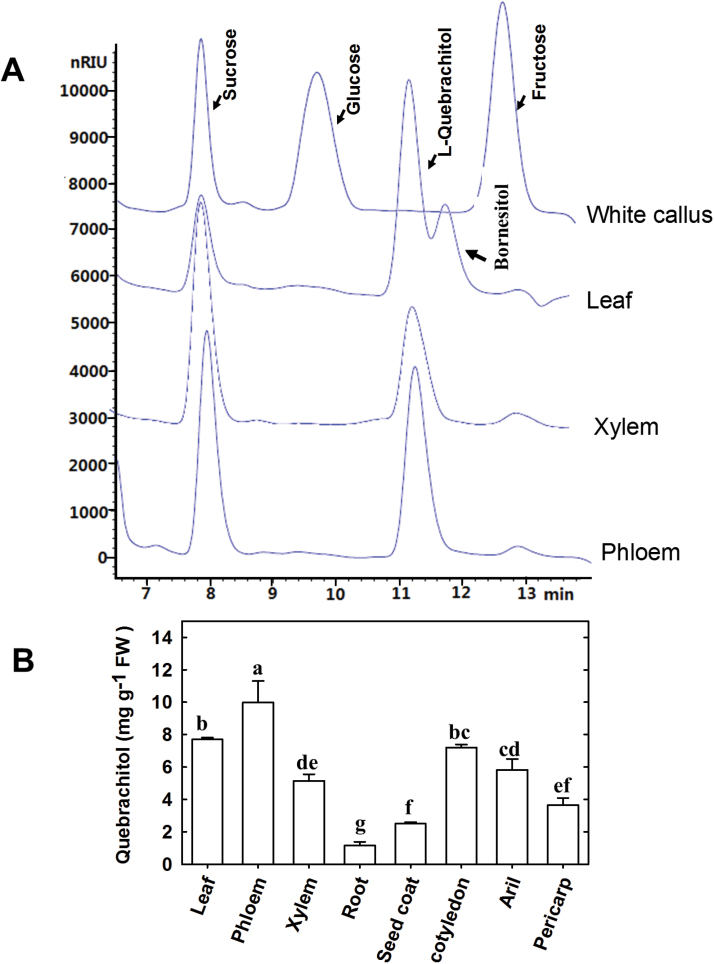
(A) HPLC chromatogram of soluble sugars in different litchi organs/tissues. (B) Concentration of quebrachitol in different litchi organs/tissues. Each data point is the mean±SE of five replicates. Different letters above the bars represent significant differences among organs/tissues (*P*<0.05) using Duncan’s multiple range test after ANOVA (n=5).

Quebrachitol was ubiquitous in the organs/tissues tested, with exception of white callus from cell suspension cultures (Fig. 2A). A peak corresponding to bornesitol was detected in leaves but not in any other tissue, including phloem and xylem, where high concentrations of quebrachitol accumulated. The quebrachitol content in 1 g of sample fresh weight (FW) ranged from 1.17 ± 0.22 to 9.99 ± 1.32 mg, being highest in the phloem and lowest in the roots (Fig. 2B). We also measured the levels of bornesitol and quebrachitol in cotyledons and seedlings (without the seed coat) during seed germination (Supplementary Fig. S3). In these samples, quebrachitol and bornesitol were found to be the major sugar derivatives, and their concentrations were constant before the leaves of the seedlings turned green and then increased as the leaves developed further.

We applied branch girdling to interrupt phloem continuity. Girdling resulted in the formation of swelled phloem callus above the girdle. Changes in sucrose and quebrachitol levels in the phloem tissues in response to girdling were then investigated (Supplementary Fig. S4). The concentration of sucrose in the phloem callus was significantly higher, and the concentration of quebrachitol was significantly lower, than in the corresponding part of the phloem in the control.

### Translocation of quebrachitol

To monitor the biosynthesis of quebrachitol and its translocation, we used isotope labeling. Radioactivity was detected in both the sucrose and quebrachitol+bornesitol fractions from the leaf extracts 2 h after feeding ^14^CO_2_ to litchi leaves (Table 1). The radioactivity in the sucrose fraction was approximately 14-fold greater than that in the quebrachitol+bornesitol fraction. These results suggested that C derived from ^14^CO_2_ fixation was incorporated more into sucrose than quebrachitol+bornesitol. In the phloem of litchi shoots that were distant to the ^14^CO_2_ feeding site, radioactivity was also detected in both the sucrose and quebrachitol fractions, but the radioactivity in the sucrose fraction was 40-fold higher.

**Table 1.** Radioactivity, including radiolabeled sucrose and quebrachitol, in leaves and phloem extracts 2 h after ^14^CO_2_ feeding

Retention time (min)	Compounds	Radioactivity(Bq)	
		Leaf extracts	**Phloem extracts**
3.0–4.3	Background	4.5 ± 0.5	4.2 ± 0.3
7.2–8.2	Sucrose	3860.6 ± 898.0	1511.0 ± 419.5
10.5–12.3	Quebrachitol (+bornesitol)^*a*^	285.4 ± 63.6	39.1 ± 6.5

^*a*^ Quebrachitol+bornesitol in leaf extracts but only quebrachitol in phloem extracts.

**Fig. 3.**
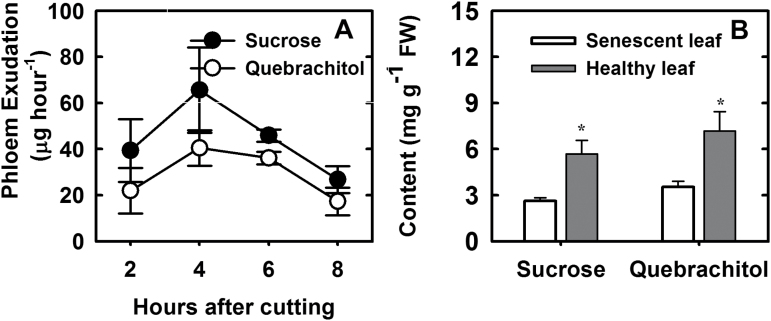
Translocation of quebrachitol. (A) Rate of phloem exudation of sucrose and quebrachitol. (B) Concentrations of sucrose and quebrachitol in healthy and senescence leaves. Each data point is the mean±SE of five replicates. *P<0.01 (*t*-test, n=5).

Phloem exudate was collected using an EDTA method (Liu *et al.*, 2012), owing to the technical challenges of directly collecting phloem sap from a woody plant such as litchi. In all the exudate samples, quebrachitol and sucrose were the predominant metabolites, with quebrachitol accounting for ~40% of the two major carbon molecules (Fig. 3A).

Senescence in green plants is a complex and highly regulated process that occurs as an integral part of development. When a leaf is no longer required, the senescence process is induced and most of the mobilizable nutrients are recycled. We measured the concentrations of sucrose and quebrachitol in both senescent and the healthy mature leaves, and observed significantly lower levels of both molecules in the senescent leaves (Fig. 3B). This result indicated that both sucrose and quebrachitol retreated from the leaf to the tree and/or converted before leaf drop.

### Quebrachitol and bornesitol in leaves and their response to stresses

**Fig. 4.**
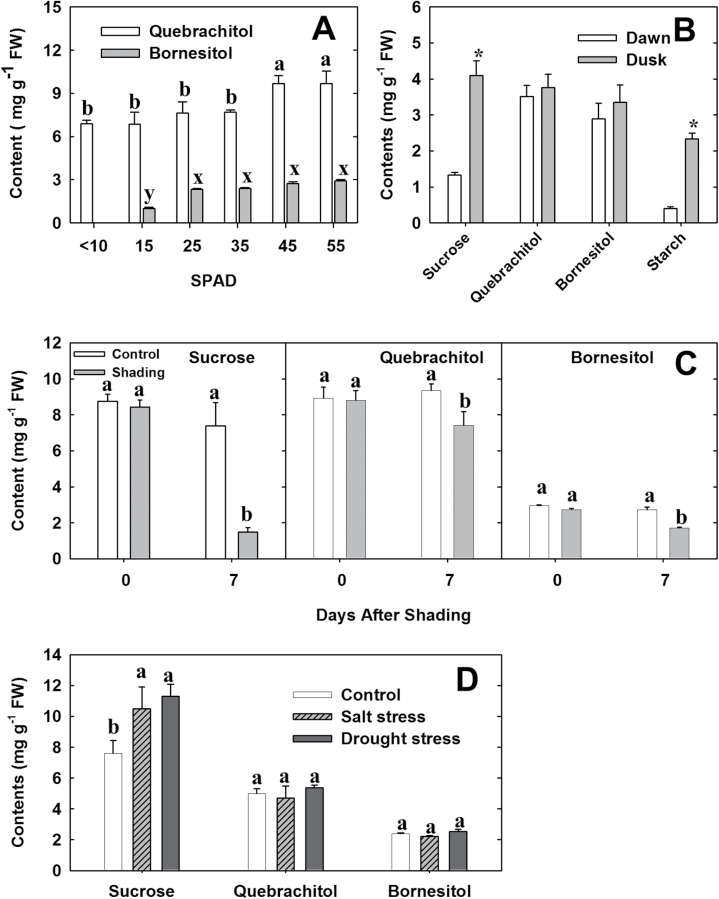
Concentrations of quebrachitol and bornesitol in litchi leaves. (A) Changes in quebrachitol and bornesitol levels during leaf development in relation to leaf SPAD values (n=8). (B) Diurnal changes in sucrose, quebrachitol, bornesitol, and starch levels in mature litchi leaves (n=8). Dawn samples were taken early in the morning, before sunrise (around 06.00 h) and dusk samples were taken in the late afternoon, before sunset (around 18.30 h). (C) Concentrations of sucrose, quebrachitol, and bornesitol in response to a prolonged period of darkness. (D) Concentrations of sucrose, quebrachitol, and bornesitol in response to salt and drought stress (n=5). Different letters above the bars of the same dependent variable (in A) or the same independent variable (in B–D) represent significant differences at *P*<0.05 using Duncan’s multiple range test after ANOVA (n=5 or 8). *P<0.01 (*t*-test, n=5).

Litchi is a typical delayed greening plant, and its fully expanded leaves continue to accumulate chlorophylls and consequently turn dark green (Hieke *et al.*, 2002). The greenness of litchi leaves, as measured using a SPAD chlorophyll meter, has been shown to be correlated with net CO_2_ assimilation; a positive net CO_2_ assimilation occurred approximately 2 weeks after the flushing stage, when the leaf SPAD value was approximately 15 (Fu *et al.*, 2014). We measured quebrachitol and bornesitol levels during leaf development and correlated the values with leaf CO_2_ assimilation (Fig. 4A). In very young leaves, when the SPAD values were <10, bornesitol was not present; it was detected as leaf development progressed, and a sharp increase was observed when the SPAD value increased from 15 to 25. Thereafter, the bornesitol concentration displayed a slight increase from 2.3 to 2.5 mg g^–1^ FW as the leaf developed toward full maturity. Quebrachitol was detected in leaves at all developmental stages, at concentrations ~7 mg g^–1^ FW before the fifth sampling stage, after which a significant increase was observed and the leaves showed maximal net CO_2_ assimilation.

During the daytime, photosynthetic carbon fixation in the leaves enables the synthesis and export of sucrose to the rest of the plant, while at night, the leaves become a net consumer of fixed carbon. Fig. 4B shows the diurnal changes in sucrose, quebrachitol, bornesitol, and starch concentrations in mature leaves from summer shoots. The sucrose and quebrachitol levels in the mature leaves from summer shoots were lower than in the leaves from autumn shoots (data in Fig. 4A), but bornesitol levels were comparable. Significantly higher levels of sucrose and starch were present at dusk than at dawn, reflecting the accumulation of sucrose and starch during the day, and the decline in their levels during the night. The diurnal fluctuations of quebrachitol and bornesitol were less pronounced, and both showed slightly higher levels at dusk than at dawn.

Potted litchi trees were used to investigate the changes in quebrachitol and bornesitol levels after a prolonged period of growth in the dark. We observed that sucrose in leaves was depleted after this period, while quebrachitol and bornesitol levels decreased to a much lesser extent (Fig. 4C).

Accumulation of methyl-inositols, such as pinitol, has been shown to be correlated with tolerance to drought and/or salinity (Vernon and Bohnert, 1992). To determine whether salt and drought stress enhanced the accumulation of methyl-inositols in litchi, we measured the concentrations of sucrose, quebrachitol, and bornesitol in leaves from salt-stressed and drought-stressed plants as well as unstressed plants. The sucrose concentration was approximately 25% (FW) higher in the plants exposed to either salt or drought stress compared with the controls (Fig. 4D). In contrast, quebrachitol and bornesitol concentrations were essentially unaffected by the stresses.

### Cloning of putative inositol methyltransferase genes

**Fig. 5.**
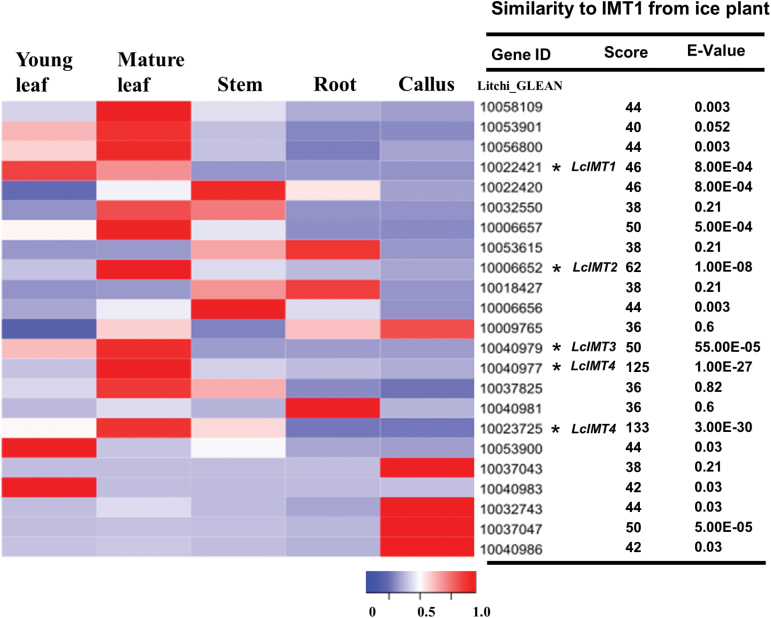
Expression patterns of 23 putative methyltransferase genes in different litchi organs/tissues that differed in quebrachitol and bornesitol levels, and their nucleotide sequence similarity to *IMT1* from ice plant (GenBank accession number M87340). Five sequences targeted for further study are indicated with an asterisk after the sequence number and designated as *LcIMT1*, *LcIMT2*, *LcIMT3*, and *LcIMT4*. Litchi CLEAN_10040977 and 10023725 were found to correspond to the same gene (*LcIMT4*).

Sequences annotated as putative methyltransferases were obtained from *in silico* analyses of the litchi genome (http://litchidb.genomics.cn/page/species/index.jsp). These sequences were compared to the previously described *M. crystallinum McIMT1* (GenBank accession number M87340; Vernon and Bohnert, 1992). Twenty-three sequences containing a methyltransferase motif were targeted for further analysis, and their expression patterns were investigated in litchi tissues or organs differing in quebrachitol and bornesitol levels (Fig. 5). The scores of their sequence similarity to *McIMT1* ranged from 36 to 125, with E-values of 0.82 to 1.0E-27 (Fig. 5). Litchi_CLEAN 10023725 had the highest similarity, followed by 10040977, 10006652, 10040979, 10006657, 10037047, 10022421, and 10022420. Among these sequences, five (Litchi_CLEAN 10022421, 10040979, 10006652, 10040977, and 10023725) were specifically expressed in leaves, and Litchi_CLEAN 10040977 and 10023725 were found to correspond to the same gene. The genes corresponding to Litchi_CLEAN 10022421, 10006652, 10040979, and 10040977 were therefore selected as putative *IMT* genes and were designated as *LcIMT1*, *LcIMT2*, *LcIMT3*, and *LcIMT4*, respectively.

### Bacterial expression and activity assay of recombinant IMT proteins

**Fig. 6.**
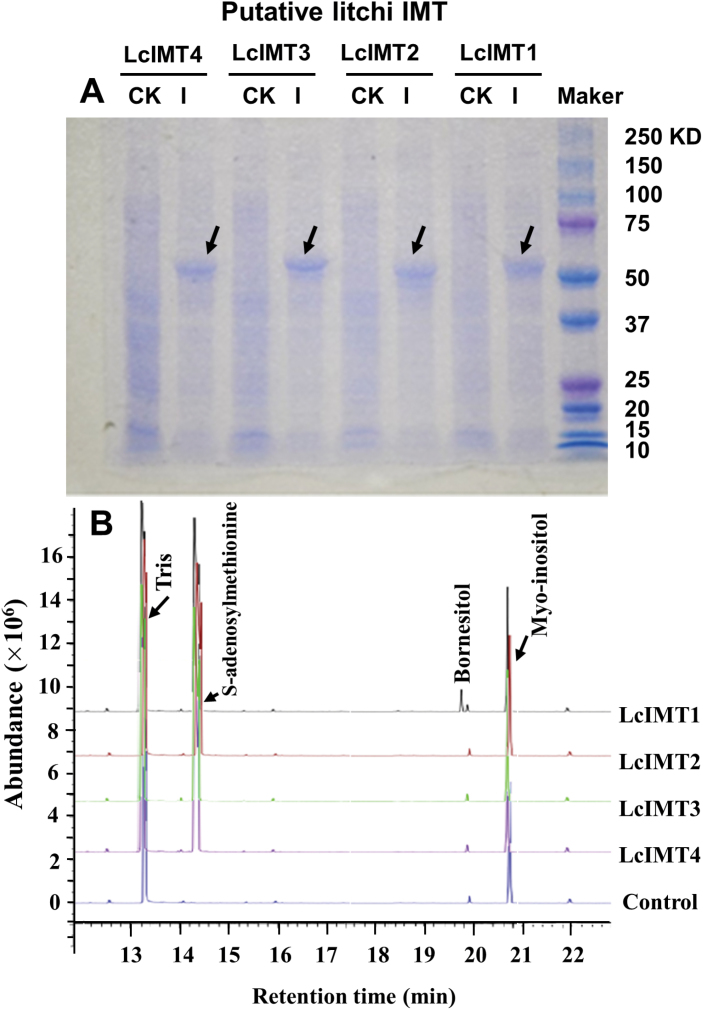
Expression of recombinant LcIMT proteins in *E. coli.* (A) Electrophoretogram of the expressed protein of four putative inositol methyltransferases. CK represents non-induced protein expression and I represents induced protein expression. (B) Inositol methyltransferase activity of the expressed proteins assayed by measuring bornesitol production using GC-MS. Peaks are identified by comparing the retention time and mass spectrum with the authentic standards or NMR-identified bornesitol.

The predicted open reading frames (ORFs) corresponding to the four putative litchi *IMT* genes were expressed in *E. coli* as ~50 kDa 6× His-tagged protein products. The recombinant proteins accumulated predominantly as inclusion bodies, with relatively low levels in the soluble fraction (Fig. 6A). The purified recombinant IMT proteins were assayed for inositol methyltransferase activity, and bornesitol production *in vitro* was confirmed by GC-MS analysis (Fig. 6B). As mentioned earlier, although different methyl-inositol isomers displayed identical mass spectrograms, they can be clearly separated and therefore identified by retention time together with their mass spectrum under GC-MS (Supplementary Fig. S2). Among the four expressed proteins, only *LcIMT1* (GenBank accession number KX886359) displayed activity in catalyzing the formation of a methyl-inositol with a retention time consistent with previously identified bornesitol (Fig. 6B). This compound was not detected in an assay with the control sample and the other expressed protein samples. We concluded that recombinant LcIMT1 was the only protein with bornesitol synthetic activity among the four proteins tested.

### *In vivo* functional analysis of *LcIMT1* using VIGS

**Fig. 7.**
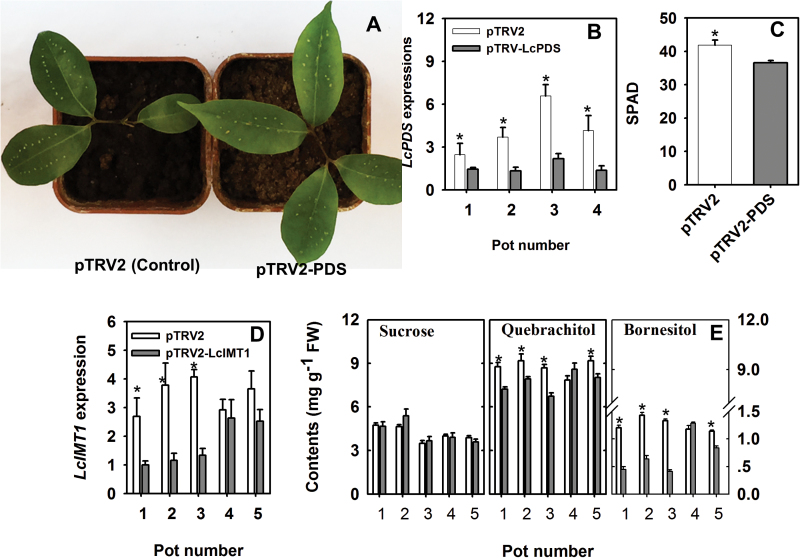
Functional verification of *LcIMT1* by suppressing its expression using VIGS. (A) Images showing virus-induced *LcPDS* silencing resulting in degreening of litchi leaves. (B) Confirmation by real-time PCR of the silencing of the *LcPDS* gene. (C) Significantly decreased SPAD values in response to virus-induced *LcPDS* silencing. (D) Confirmation by PCR of the silencing of the *LcIMT1* gene. (E) Quebrachitol and bornesitol levels, showing a significant decrease in response to virus-induced *LcIMT1* silencing. Each data point is the mean±SE of four replicates. *P<0.01 (*t*-test, n=4).

VIGS is a powerful and rapid tool to achieve loss of function of the targeted gene, and has been successfully applied in many plant species (Velásquez *et al.*, 2009). For example, VIGS has been used to induce silencing of the expression of a phytoene desaturase (PDS, EC 1.3.99.30) gene in the leaves of litchi, resulting in a photobleached phenotype, and to induce the silencing of *LcUFGT1* in fruits, resulting in loss of pigmentation (Li *et al.*, 2016). In the present study, we validated the use of VIGS in combination with an agroinfiltration method using *A. tumefaciens* strain GV3101 harboring TRV1 mixed with GV3101 containing the TRV2 vector with the *LcPDS* gene inserted. Ten days after agroinfiltration, we observed bleaching, a decrease in *LcPDS* expression, and a decrease in the SPAD value in leaves (Fig. 7A–C).

VIGS was then used to silence the expression of *LcIMT1* in mature leaves. Twelve days after infiltration, we observed a decrease in *LcIMT1* expression. Consistent with the reduced expression of *LcIMT1*, the concentrations of bornesitol and quebrachitol in leaves of plants infiltrated with TRV1 and TRV2 containing the *LcIMT1* insert (pot numbers 1, 2, 3, and 5) were significantly lower than those in the control leaves infiltrated with an empty vector, with a relatively greater drop in the level of bornesitol than quebrachitol (Fig. 7D, E). In contrast, the sucrose concentrations in *LcIMT1*-silenced leaves were generally similar to those in control leaves (Fig. 7E).

### *In silico* analysis of *LcIMT1* and other methyltransferases

The sequence of *LcIMT1* was compared with that of the previously reported *McIMT1* cDNA (Vernon and Bohnert 1992). The complete nucleotide sequence of *LcIMT1* is predicted to have an ORF of 1059 bp, which encodes a 353 amino acid protein and is 26 bp shorter than the sequence of *McIMT1* (1095 bp, encoding a 365 amino acid protein) (Supplementary Fig. S5). Sengupta *et al.* (2008) isolated and characterized *PcIMT1* (GenBank accession number EU240449), an inositol methyltransferase from a halophytic wild rice species (*Porteresia coarctata*), which was shown to regulate pinitol synthesis under abiotic stresses. The identical protein product (365 amino acids long) shows only two amino acid deviations from that of McIMT1, with G in place of D16 and V in place of I237 (Sengupta *et al.*, 2008). Although LcIMT1 has the two *O*-methyltransferase domains, SAM-binding motifs, and the dimerization domain that are common among plant methyltransferases, it has much lower sequence similarity with the previously reported McIMT1 proteins than PcIMT1.

## Discussion

### Long-distance transport of quebrachitol in litchi

High accumulation of methyl-inositols has been reported in a number of plant species (Vernon *et al.*, 1993; Wanek and Richter, 1997; Sengupta *et al.*, 2008; Negishi *et al.*, 2015). Their chemical properties and metabolically inert nature make them ideally suited to serve as compatible solutes under stress conditions, and as phloem-translocated compounds. In this study, we found that quebrachitol was ubiquitous in litchi organs and tissues, with especially high levels in leaves, phloem, and xylem (Fig. 2), where levels were comparable to or even higher than those of sucrose.

The role of quebrachitol as a translocated photoassimilate has not been described, although its isomeride, pinitol, has previously been suggested to be translocated in soybean, based on its presence in phloem sap and its patterns of accumulation (Campbell and Binder, 1984; Guo and Oosterhuis, 1995; Gomes *et al.*, 2005). In the present study, owing to the lack of commercially available [^14^C] quebrachitol to study its translocation, we fed ^14^CO_2_ to litchi leaves, combined with fraction collection by HLPC and detection of radioactivity in the fractions. The results clearly showed that ^14^C was incorporated into both quebrachitol and sucrose in the leaves, and that quebrachitol was subsequently loaded into the phloem together with sucrose (Table 1). The radioactivity in the quebrachitol fraction was much lower than that of the sucrose fraction, suggesting that more fixed carbon was incorporated into sucrose. Interestingly, the standing concentration of quebrachitol in the leaves was similar to that of sucrose. This discrepancy might be caused by the difference in the time taken for the synthesis of sucrose and quebrachitol, as sucrose biosynthesis from CO_2_ involves fewer biochemical reactions. The detection of polyols in phloem sap is often used as evidence of long-distance transport (Noiraud *et al.*, 2001*a*). An analysis of phloem exudates revealed that quebrachitol represented ~40% of the constituent carbohydrates (Fig. 3A). Since the levels of quebrachitol and sucrose were detected constantly during the 8 h experiment, we concluded that phloem loading and transport of molecules from companion cells into the sieve elements may continue during exudation until the loss of vitality.

The synthesis of methyl-inositols occurs mainly in source leaves (Vernon and Bohnert, 1992; Guo and Oosterhuis, 1995; Chiera *et al.*, 2006). The absence of the corresponding synthetic enzymes in the sink organs where methyl-inositols were detected (Vernon and Bohnert, 1992) further suggests that long-distance transport of methyl-inositols occurs. Quebrachitol was detected in litchi sink organs/tissues, such as arils, seeds, and roots (Fig. 2).

Taken together, these results support the hypothesis that quebrachitol is a translocated compound.

### The inert nature and physiological role of quebrachitol

Quebrachitol is a major sugar derivative in litchi leaves, with concentrations (5–9 mg g^–1^ FW) that are comparable to, or even higher than, those of sucrose (4–8 mg g^–1^ FW) (Fig. 4). It is well established that carbohydrates, especially sucrose and starch, undergo marked diurnal changes in concentration in leaves (Kelly, 2006). In the present study, the diurnal fluctuations of quebrachitol and bornesitol concentrations were much less apparent than those of sucrose and starch (Fig. 4B). Slight decreases in leaf quebrachitol and bornesitol concentrations were observed in leaves at dawn (i.e. after nighttime), in contrast to the significant depletion of foliage sucrose and starch during the night (Fig. 4C). Furthermore, the concentrations of quebrachitol and bornesitol remained unchanged during seed germination and before the leaves turned green (Supplementary Fig. S3). These results indicate that quebrachitol is probably an inert source of reserve energy, which is consistent with a previous study of white clover (*Trifolium repens*) and soybean (Smith and Phillips, 1982). However, the significant decrease in quebrachitol in senescent leaves suggested that quebrachitol can be recycled or converted (Fig. 3B). In addition, the significant decrease in quebrachitol and the significant increase in sucrose in girdling-induced phloem callus imply that quebrachitol can be converted into other sugars and then utilized by plants (Fig. 4). Although the degradation pathway of inositol is well established, the degradation of methyl-inositol has not been reported. This is worth further study.

The inert nature of methyl-inositols makes them valuable compatible solutes, and various forms, particularly pinitol, have been associated with salinity and other stresses in a diverse range of plants, including maritime pine (*Pinus pinaster*) (Nguyen and Lamant, 1988), ice plant (Vernon and Bohnert, 1992), halophytic wild rice (Sengupta *et al.*, 2008), soybean (Guo and Oosterhuis, 1997), and rice bean (*Vigna umbellata*) (Wanek and Richter, 1997). Pinitol levels were reported to be relatively low in the leaves of unstressed ice plants, but to increase approximately 3-fold in salt-stressed leaves (Vernon and Bohnert, 1992). Additionally, a 10-fold increase in pinitol level was observed in the leaves of halophytic rice subjected to salt stress (Sengupta *et al.*, 2008). However, we observed that the foliage concentrations of quebrachitol and bornesitol were essentially unchanged in response to salt or drought stresses (Fig. 4D). Moreover, in unstressed litchi leaves, quebrachitol was present as a major sugar derivative (Fig. 4), suggesting that quebrachitol accumulation in litchi is not associated with stress. The role of methyl-inositols in osmotic stress is still not well established. For example, soybean produces large amounts of pinitol (~30 mg g^–1^ dry weight), but is still sensitive to drought, cold, and heat (Chiera *et al.*, 2006).

Sucrose levels in litchi leaves ranged from 4 to 8 mg g^–1^ FW (12–23 mM), which is low compared with those of other woody plants. Rennie and Turgeon (2009) surveyed the leaf sugar concentrations of 45 species in 36 dicotyledonous families. Woody plants, such as *Prunus laurocerasus*, *Corylus colurna*, *Juglans ailantifolia*, and *Platanus occidentalis*, had much higher leaf sugar levels (>50 mM) and therefore higher osmolalities than most herbaceous plants, such as *Chrysanthemum rubellum*, *Vinca minor*, *Citrullus lanatus*, and *Nicotiana rustica* (<20 mM). According to Fu *et al.* (2011), trees must offset low hydraulic conductance with high concentrations of foliar transport sugars, which provide the motivating force for sugar diffusion and render active phloem loading unnecessary. This reduces inventory costs and significantly increases growth potential. Litchi is a large woody plant, and the high concentrations of quebrachitol (5–9 mg g^–1^ FW; 26–46 mM) may represent an important osmolyte solute to compensate for the lower sucrose concentration, therefore not only facilitating water transport but also maximizing carbon use efficiency and growth.

Higher plants show taxonomic diversity in their preferential accumulation of certain compatible solutes, such as sorbitol in members of the Rosaceae and mannitol in species in the Apiaceae. The presence of higher levels of quebrachitol might be a taxonomic trait of plants belonging to the Sapindaceae, since this is also the case for longan (*Dimocarpus longan*) (Wang *et al.*, 2013), rambutan (*Nephelium lappaceum*) (data not shown), and other species in this family (Kiddle, 1995; Díaz *et al.*, 2008). The ability of the Sapindaceae to produce quebrachitol might have evolved early in the evolution of this ancient family. High quebrachitol but low sucrose concentrations in tissues of litchi might represent a unique carbon metabolic strategy that renders this species less attractive to pests and diseases, thus reducing the risk of biotic stresses while maintaining osmolality. Litchi is one of the most important subtropical fruit crops, with a history of being cultivated for thousands of years. In contrast to fruit crops from other families, litchi is less attractive to, and less damaged by, pests and diseases. In apple, the progressive expression of resistance to fire blight with leaf age along an apple shoot parallels an increase in sorbitol concentration (Suleman and Steiner 1994). Furthermore, the response of global transcript levels in apple leaves to decreased sorbitol synthesis suggests that sorbitol plays a role in biotic stress (Wu *et al.*, 2015). The role of quebrachitol in biotic stress in litchi will be the target of future studies.

### Biosynthetic pathway of quebrachitol

Most cyclitols are synthesized from the ubiquitous plant sugar alcohol myo-inositol (Loewus and Dickinson, 1982). One of the best studied cyclic sugar alcohols is pinitol, which is thought to be synthesized in a two-step pathway involving the methylation of myo-inositol, followed by epimerization of the methylated intermediate (Loewus and Dickinson, 1982; Dittrich and Korak, 1984). Ononitol was reported to be the major soluble carbohydrate constituent in the leaves of tobacco transformed with the inositol *O*-methyltransferase gene (*McIMT1*) from ice plant (Vernon *et al.*, 1993), and ononitol levels increased 10- to 80-fold in *McIMT1*-overexpressing soybean embryogenic tissues compared with non-transgenic tissues (Chiera *et al.*, 2006). These results suggested that McIMT1 catalyzes the formation of the methylated intermediate, ononitol.

In the present study, bornesitol but not ononitol was detected in litchi leaves with net CO_2_ assimilation (reflected by SPAD values) and its concentration progressively increased during leaf development (Supplementary Figs S1 and S2, Fig. 4A). The concentration of bornesitol also increased during leaf maturation in litchi seedlings (Supplementary Fig. S3). These results suggest that inositol methylation takes place in the leaves and that the production of methyl-inositol is closely associated with carbon fixation. The presence of bornesitol but not ononitol in the leaves indicates a different biosynthetic pathway to that of pinitol.

**Fig. 8.**
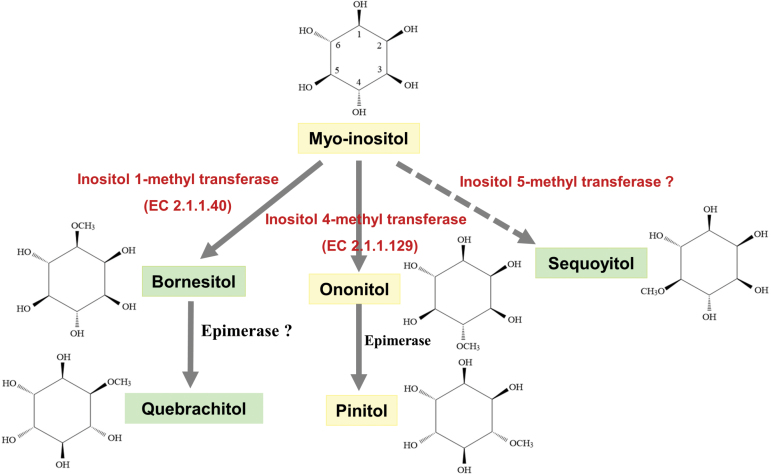
Simplified scheme of the putative biosynthetic pathway of five methyl-inositols, based on previously reported results and data from the present study.

The production of ononitol (4-*O*-methyl-myo-inositol) involves the methylation of myo-inositol by inositol 4-*O*-methyltransferase (Dittrich and Korak, 1984). Inositol 1-*O*-methyltransferase has been identified to catalyze the formation of bornesitol (1-*O*-methyl-myo-inositol) (Schomburg *et al.*, 2006). In litchi, the methylation of myo-inositol by inositol 1-*O*-methyltransferase, followed by epimerization of bornesitol, the transient methylated intermediate, might be responsible for the production of quebrachitol (Fig. 8). According to Bieleshi and Briggs (2005), bornesitol was the widely distributed polyol in various species and genera of the Proteaceae. Some species they tested had bornesitol as the only polyol, while other species contained significant amounts of quebrachitol as well as bornesitol. Ononitol was the only methyl-inositol that accumulated in the leaves of ice plant in response to salinity stress (Nelson *et al.*, 1998). Soybean accumulated pinitol as the major methyl-inositol, with only traces of ononitol (Streeter *et al.*, 2001). The methyl-inositol profile in plants seems depend on the types of inositol methyltransferase and the presence of an epimerization system. Inositol 3-*O*-methyltransferase is another reported inositol methyltransferase, which catalyzes the formation of 3-*O*-methyl-myo-inositol (Schomburg *et al.*, 2006). However, there has been no report of the identification of 3-*O*-methyl-myo-inositol in plants. Sequoyitol (5-*O*-methyl-myo-inositol) is a more common methyl-inositol in plant species, especially members of the families Taxaceae, Pinaceae, Taxadiaceae, and Cupressaceae (Plouvier, 1960). Unfortunately, the enzyme responsible for sequoyitol biosynthesis has not been characterized. According to the rules of ononitol and bornesitol synthesis, we deduce that inositol 5-*O*-methyltransferase might be the inositol methyltransferase responsible for the formation of sequoyitol (Fig. 8).

### *LcIMT1* is responsible for the biosynthesis of bornesitol

Four putative *LcIMT* genes were targeted after searching the litchi genome for predicted methyltransferase sequences, analyzing their similarity with the previously described *McIMT1* gene, and investigating their expression patterns (Fig. 5). After expression of the corresponding recombinant proteins in *E. coli*, we determined that only LcIMT1 had detectable inositol methyltransferase activity (Fig. 6B), suggesting that it functions as a methyltransferase involved in the accumulation of bornesitol. This conclusion was validated by the effect of down-regulation of *LcIMT1* through VIGS, which resulted in significantly lower concentrations of bornesitol (Fig. 7E).

As far as we know, *McIMT1* and *PcIMT1* are currently the only reported *IMT* genes. Their translated protein products (both 365 amino acids long) show 99% sequence similarity, with variation at only two amino acids (Sengupta *et al.*, 2008). The reaction product catalyzed by both McIMT1 and PcIMT1 is ononitol (Vernon and Bohnert, 1992; Sengupta *et al.*, 2008), while the product of LcIMT1 is bornesitol (Fig. 2, Fig. 4). We note that the translated protein LcIMT1 is 353 amino acids long, which is 12 amino acids shorter than McIMT1 and PcIMT, and that these ‘missing’ amino acids are mainly located at the N-terminus (Supplementary Fig. S5). Although highly conserved in the functional domains, LcIMT1 has low sequence similarity with the previously reported IMT proteins (Supplementary Fig. S5). The sequence differences might be associated with the different hydroxyl methylation site on inositol. Identification of the gene/enzyme responsible for epimerization of the methylated intermediate will be a valuable next step in the elucidation of the quebrachitol biosynthetic pathway in litchi.

## Supplementary data

Supplementary data are available at *JXB* online.

Table S1. Primers used for real-time PCR assay of putative methyltransferases.

Table S2. Primers used for amplifying the coding regions of *LcIMT* genes.

Fig. S1. HPLC chromatogram of four methyl-inositol standards and the collected fraction of 10.5 to 12.3 min eluate from the litchi leaf.

Fig. S2. GC-MS total ion chromatogram of the *N*-methyl-*N*-trimethylsilyl-trifluoroacetamide derivatives of the four methyl-inositol standards and the leaf putative methyl-inositol in litchi.

Fig. S3. The developmental changes in quebrachitol and bornesitol levels during seed germination.

Fig. S4. Quebrachitol and bornesitol concentrations in the girdling-induced phloem callus and control phloem tissue.

Fig. S5. Protein alignment of four LcIMTs with McIMT1 and PcIMT1, functionally characterized inositol methyltransferase proteins.

Supplementary Tables and Figures
