# Supplementary material for: Biosynthesis of quebrachitol, a transportable photosynthate, in Litchi chinensis
Source: J Exp Bot. 2017 Dec 21;69(7):1649–61. doi: 10.1093/jxb/erx483 (PMC5889025; doi:10.1093/jxb/erx483)
Supplement: Supplementary Tables and Figures [file erx483_suppl_supplementary_tables_and_figures.pdf]

Table S1. Primers used for real-time PCR assay of putative methyl transferases.

Sequences	Forward primer (5' to 3')	Reverse primer (5'to 3')	Product size (bp)
Litchi_GLEAN_10023725	ATCCAAGGTAGTATTCCA	AATCATCTTCAGTCCTCT	77
Litchi_GLEAN_10040977	GAGAGGACTGAAGATGATT	AAGCACAGCATACTACTT	76
Litchi_GLEAN_10006652	ACATTGCTTGAGGCTATTGA	GCGTGTCTGGAACTATCG	89
Litchi_GLEAN_10006657	TACAAGTCCATTCCAGAT	GAGTCATCATAAGCATATCA	118
Litchi_GLEAN_10040979	GAGACTTGTTCGTTAGTATTCC	TGCTCATCACTCCAATCAT	79
Litchi_GLEAN_10037047	TGAGCAAGAACGGAAGAA	ATAATGGATGGTATAGCAAGGA	84
Litchi_GLEAN_10022421	CTATAATGGCGTGTCTCA	ATCTTCTTCAGTCCAATCA	103
Litchi_GLEAN_10022420	TGAGCAAGAACGGAAGAA	ATAATGGATGGTATAGCAAGGA	83
Litchi_GLEAN_10006656	GAGGCAATACTTCCAGTT	TCATCATAAGCACATCCAT	76
Litchi_GLEAN_10056800	AAACATCAGCAAGCCATT	CCATCACAGCAAGCATAA	119
Litchi_GLEAN_10053900	TATAGTCTGAATCCTGTCTC	ACCTTGTCCTGATGTAAG	89
Litchi_GLEAN_10058109	AGGCAATAGTTCCAGACA	TCATCATAATCGCATCCATT	75
Litchi_GLEAN_10032743	ACATTGCTTGAGGCTATTGA	GCGTGTCTGGAACTATCG	89
Litchi_GLEAN_10040986	TGTTCATTAGTGTTCCAA	CTTCATAGCAGTTCTTCA	105
Litchi_GLEAN_10040983	TGTTCATTAGTGTTCCAA	CTTCATAGCAGTTCTTCA	105
Litchi_GLEAN_10053901	TACAAGTCCATTCCAGAT	GAGTCATCATAAGCATATCA	118
Litchi_GLEAN_10018427	CGTTGGAGGAGACTTGTT	ATCTTCAGGCATTCTTCGT	96
Litchi_GLEAN_10053615	GTTGGAGGAGACTTGTTC	ATCTTCAGGCATTCTTCG	95
Litchi_GLEAN_10037043	GCATAGGATCGTTAGTTG	TTGATGTGAGGATGAGAT	79
Litchi_GLEAN_10032550	TTGTTCGTAGATGTTATGAT	TTAATGTGAGGGTATTTGG	157
Litchi_GLEAN_10037825	GACTTATGATGTGTATGAT	ATTCTTCCTCCTTGTATA	137
Litchi_GLEAN_10009765	AGAATGAACAATCTCTACAATG	AGACCACCACCAATATCC	122
Litchi_GLEAN_10040981	TATCCTTACACCTACCAATG	TATCCGCAACAATGAGAA	86

Table S2. Primers used for amplifying the coding regions of LcIMT genes.

Sequences	Forward primer (5' to 3')	Reverse primer (5'to 3')	CDS (bp)
LcIMT1	ATGGGATCGGTAGAAGAAGAG	TCACTGTGGATAGGCCTCAA	1062
LcIMT2	ATGAATTCTTTAGTGGAAAACCAAC	TTATTTGTACAACTCCATAATACCG	915
LcIMT3	ATGGATTCAACACAAATCTCAGATC	TCAAGTTTTCTTCAGAAATTCCATT	1086
LcIMT4	ATGACTCCGACCAACGTCTC	TCAAGCCTTCTTCAGAAATTC	1074

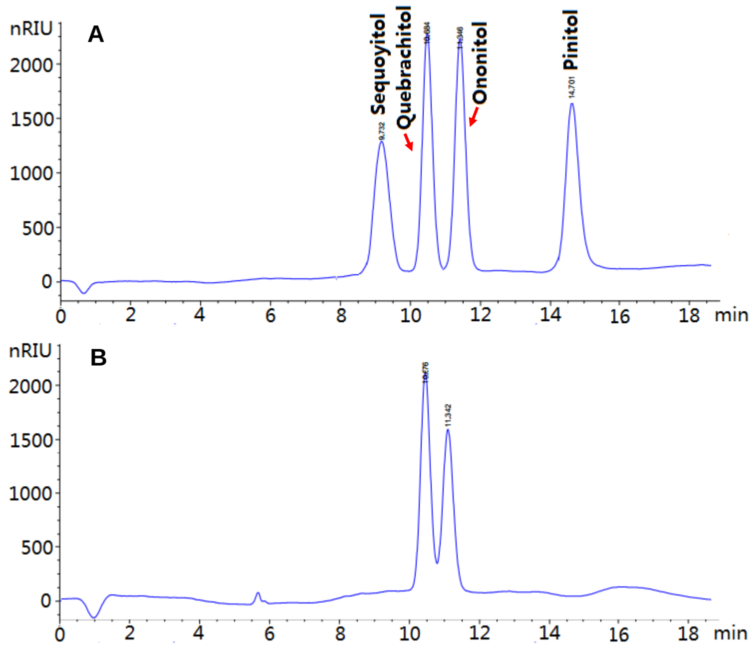

Fig. S1. High-pressure liquid chromatography (HPLC) chromatogram of four methyl-inositol standards (A) and the collected fraction of 10.5 to 12.3 min eluate from the litchi leaf (B).

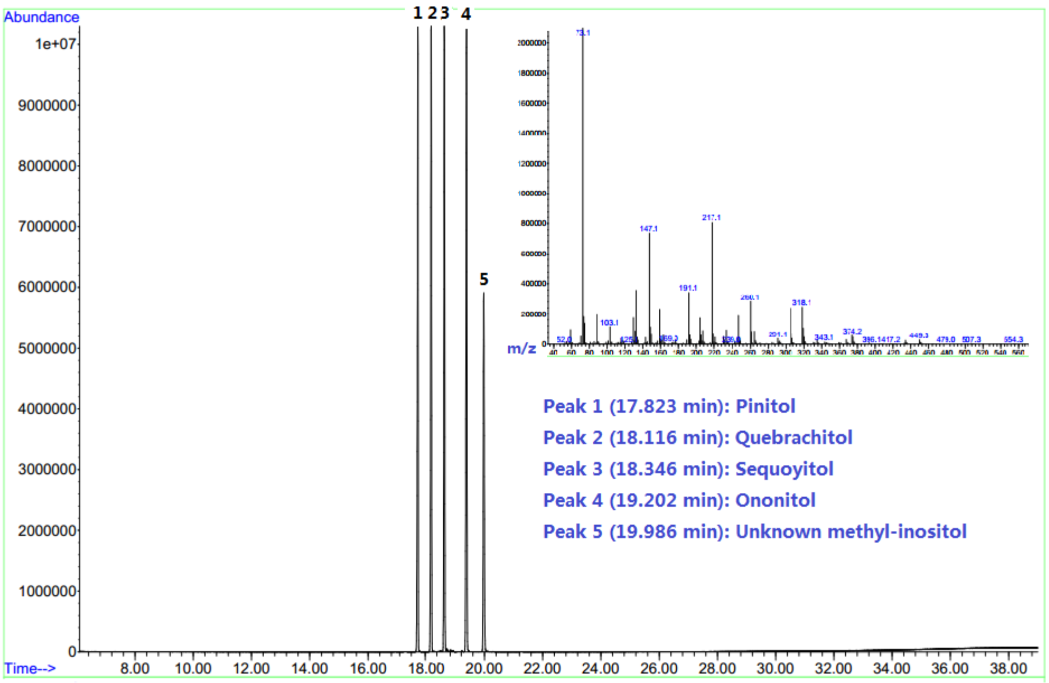

Fig. S2. Gas chromatography-mass spectrometry (GC-MS) total ion chromatogram of the N-methyl-N-trimethylsilyl-trifluoroacetamide (MSTFA) derivatives of the four methyl-inositol standards and the leaf putative methyl-inositol in litchi. The inset appearing at the top right corner is the mass spectrogram of peak 5, which is identical with the four methyl-inositol standards.

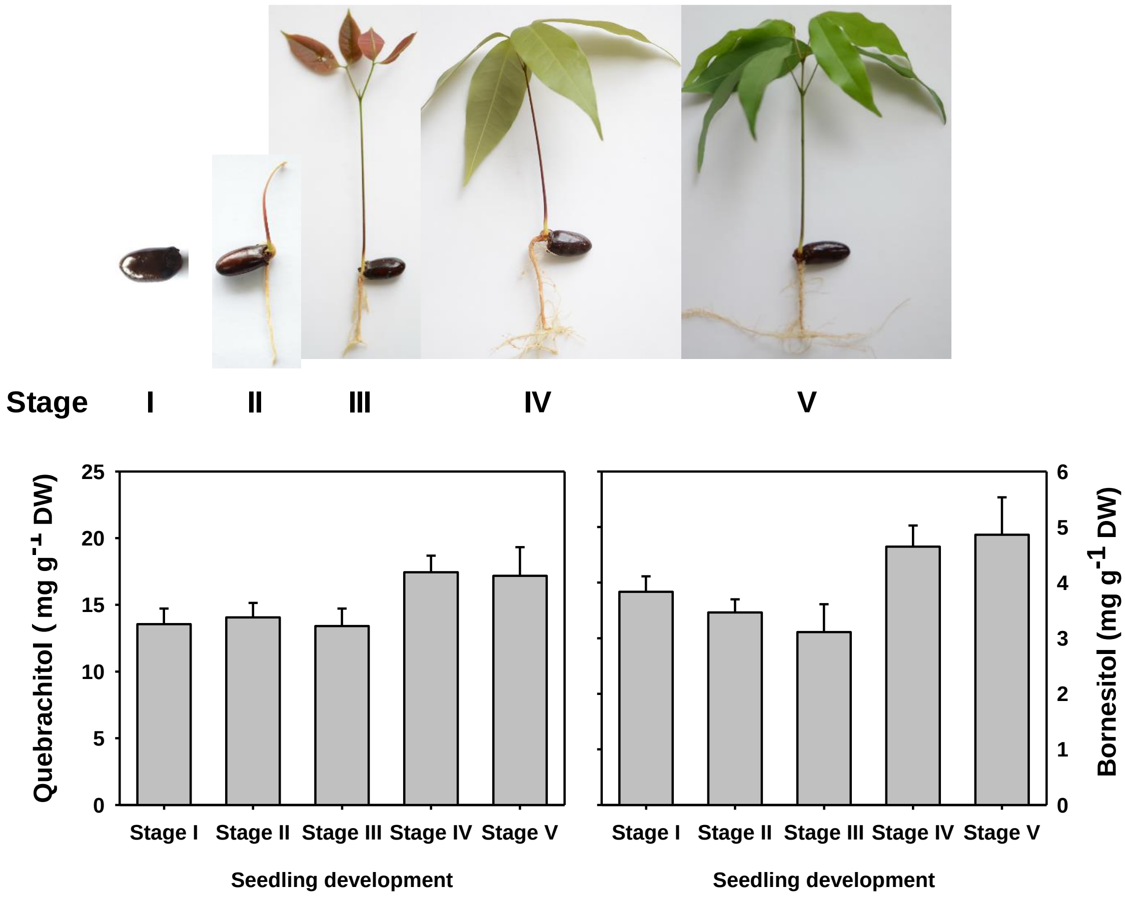

Fig. S3. The developmental changes in quebrachitol and bornesitol levels during seed germination. Vertical bars represent standard error of the mean (n = 5).

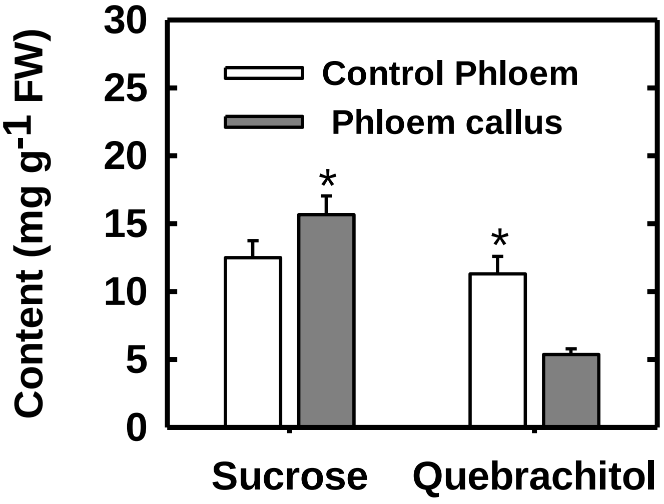

Fig. S4. Quebrachitol and bornesitol concentrations in the girdling-induced phloem callus and control phloem tissue. The asterisk (*) represents significant difference at $p<0.01$ using T-test (n=10).

```
LcIMT1  : -------MGSVEEERAALLRGQAEVWDIMFAFADSIALKAAVELRVPDIVHSHG--GPITLTQIASKIDATSPDIS-YIA
LcIMT2  : -MNSIVENQLKPNKEEDEKEAFSYATQLATGVAINMSLQSAIELGVFDIIAKAGPGASLSASEIASQLSS----------
LcIMT3  : ----MDSTQISDRDQEADY---LYAMRLASGSVLPMVLKSAIELDVLEIIARAGPGGSMSPNEIASHLPTSNPDAPIMLD
LcIMT4  : ----MTPTNVSD--EEAN----LFAMQLTSASVLPMVLKSAIELDLLEIIAKAGSGAHLSPTEVASQLPTTNPDAPVMLD
McIMT1  : MTTYTNGNYTQPKTLDKDEQIAGLAVTLANAAAFPMILKSAFELKILDIFSKAGEGVFVSTSEIASQIGAKNPNAPVLLD
PcIMT1  : MTTYTNGNYTQPKTLGKDEQIAGLAVTLANAAAFPMILKSAFELKILDIFSKAGEGVFVSTSEIASQIGAKNPNAPVLLD

LcIMT1  : RVMRLIVRKGVFTADYPS---SGGEPQYGATDVSKWLLQDAD-LTIAPMLIMENDPVQSDPWHYIGNCVKEGGVPFEKVH
LcIMT2  : -----------VTNRIK-----------------------------------KSQIIM---FQLKDAVLEGGVPFDRVH
LcIMT3  : RMLRLIASYSILTCSLRSLPDGKVERLYGIAPVCKYFTKNEDGVSLSAFSLLGQDKVVMESWYCFKDAVLEGGVPFEKTH
LcIMT4  : RILRLIASYSVLTCSIRTLPDAKVERLYGLGPVCKFLTKNEDGVSISDLSIMNQDKVIMESWYHLKDAVLEGGIPFNKAY
McIMT1  : RMLRLIASHSVLTCKLQK-GEGGSQRVYGPAPLCNYIASNDGQGSLGPLIVLHHDKVMMESWFHINDYILEGGVPFKRAH
PcIMT1  : RMLRLIASHSVLTCKLQK-GEGGSQRVYGPAPLCNYIASNDGQGSLGPLIVLHHDKVMMESWFHINDYILEGGVPFKRAH

LcIMT1  : GEDFWYLIPKKPKFEKLFYAAMACTSKVVMRAVVSECKDVFANIGSIVDVAGGIGGAISEVVKAYPHIKGINFDQPHVVA
LcIMT2  : GTNAFEYPGMDSRFNDVFNKAMYNHTTIVIKKILEVYRG-FENLKQIVDVGGGLGVTIKAITAKYPHIKGINFDLPHVIQ
LcIMT3  : GMAWFECLSINPTFNKIFNNAMASISTITLKKILETYKG-FEGLKSVIDVGGGTGAAINMIISIYPTIRGINFDLPHVIK
LcIMT4  : GMTAFDYHGTDPRFNKVFNNGMSSHSTITMKKILETYKG-FEGLKSIVDVGGGTGATVNMIVSLYPSIKGINFDLPHVIE
McIMT1  : GMIQFDYTGTDERFNHVFNQGMAHHTIIVMKKLLDNYNG-FNDVKVLVDVGGNIGVNVSMIVAKHTHIKGINYDLPHVIA
PcIMT1  : GMIQFDYTGTDERFNHVFNQGMAHHTIIVMKKLLDNYNG-FNDVKVLVDVGGNIGVNVSMIVAKHTHIKGINYDLPHVVA

LcIMT1  : TAPAYNGVSHVGGNMFDTIPK-ADAVMMKWILHDWTEEDGIKILKNCRKAIPEKNGKVILIDFVVKEDGNKKYG-DIDVR
LcIMT2  : HASHYPGVEHVGGDMFESVPE-SDAIFLKWVLHDWSDEHCLRLLKNCYKAIP-DDGKVIIVEAIVPDTPEATAATKATSQ
LcIMT3  : DAPSYPGVTHVEGDLFVSIPKGGDAIFLKWICHDWSDEHCLKLLKNCHEALP-DDGKMFVCEWILPAALDPSLKSNVVLH
LcIMT4  : DAPAYPGVEHVGGDMFVSVPK-ADAIFMKWICHDWSDEHCLKFLKKCYEALP-KNGKVIVCECVLPVTPDPSLGSKVVFH
McIMT1  : DAPSYPGVEHVGGNMFESIPQ-ADAIFMKWVLHDWSDEHCVKILNKCYESIA-KGGKIIIVESLIPVIPEDNLESHMVFS
PcIMT1  : DAPSYPGVEHVGGNMFESIPQ-ADAIFMKWVLHDWSDEHCVKILNKCYESIA-KGGKIIIVESLIPVIPEDNLESHMVFS

LcIMT1  : FDIVMLAHNKGGRERTEEEWKKLLGAGGFPRYRIIKVPSVLHVIEAYPQ-
LcIMT2  : IDAIMMTQNPGGKERTRYEFLALATGAGFSGIRFECFACNFGIMELYK--
LcIMT3  : FDCIMMSSFKDGKERTEDEFKALAKAAGFQGFQIVRCAPAIYIMEFLKKT
LcIMT4  : IDCIMLAHNPGGKERTEDDFRALAKGAGFQGFKVVCCAFNTYIMEFLKKA
McIMT1  : LDCHTIVHNQGGKERSKEDFEALASKTGFSTVDVICCAYDTWVMELYKK-
PcIMT1  : LDCHTIVHNQGGKERSKEDFEALASKTGFSTVDVICCAYDTWVMELYKK-
```

Fig. S5. Protein alignment of four LcIMTs with McIMT1 and PcIMT1, functionally characterized inositol methyl transferase proteins. Four conserved *O*-methyl transferase function domains are shaded.
